# Significant Day-time Ionospheric Perturbation by Thunderstorms along the West African and Congo Sector of Equatorial Region

**DOI:** 10.1038/s41598-020-65315-3

**Published:** 2020-05-21

**Authors:** B. O. Ogunsua, A. Srivastava, J. Bian, X. Qie, D. Wang, R. Jiang, J. Yang

**Affiliations:** 1 Key Laboratory for middle Atmosphere and Global Environment Observation (LAGEO), Institute of Atmospheric Physics (IAP), Chinese Academy of Science, Beijing, China; 2 Department of Physics, Federal University of Technology, Akure, Nigeria; 3 College of Earth and Planetary Sciences, University of Chinese Academy of Sciences, Beijing, China

**Keywords:** Atmospheric dynamics, Space physics

## Abstract

The equatorial Congo has been recognized as the most active lightning chimney region in the Globe. Although the perturbation of tropospheric thunderstorms on the lower ionosphere has been noticed in the middle latitudes through their transient lightning electric fields or convective gravity waves, the effects on equatorial ionosphere and the horizontal extent of this perturbation remains a mystery because of the difficulties in extracting the effects due to the sporadic nature of the equatorial ionosphere. Here we present observational results showing solid evidence of deviations in ionospheric total electron content (TEC) and its direction of propagation associated with thunderstorms using the method of polynomial filtering, by utilizing the TEC measured from equatorial Global Positioning System (GPS) Receiver stations along the West African region-Congo Basin. The TEC deviations due to the thunderstorms were found to be mostly propagated in a specific direction from the point of the event, with the highest absolute peak TEC at ~±1.5 TECUs. The internal dynamics of the equatorial ionosphere have been found to be suppressed by large thunderstorm effects during the daytime, with negligible impact at night.

## Introduction

The ionosphere is a system that can be dynamically perturbed, due to the direct external influences from solar dynamics and activities (such as geomagnetic storms and solar radiations events). It can also be influenced by the internal forcing dynamics of the earth’s neutral atmosphere (including the effect of the thermosphere, mesosphere, stratosphere and troposphere)^1^. The influence of the solar activities on the ionosphere has been vastly investigated and explored from different perspectives, however the effect of lower atmosphere on the ionosphere requires more investigations. In particular, a thorough inquisition into the effect of thunderstorms on the dynamics of equatorial ionosphere with the use of TEC data measured above thunderstorm, which has not been carried out would have great impact on our knowledge of the ionospheric dynamics during these events, especially for the ionosphere around the equatorial/low latitude region.

A number of evidences have linked the D-layer heating to thunderstorm’s quasi-electrostatic fields^2–4^. Recently, an attempt was made to model the perturbations in the ionosphere due to resultant heating of the lower atmosphere resulting from thunderstorm and it was found that there are some consistencies in the modelled result obtained compared with experimental results. They implied that the result suggests that the VLF perturbations in the ionosphere might have resulted from the modified quiescent heating of the low ionosphere^4^. Evidence of possible direct heating of the D-layer due to electromagnetic energy from lightning pulses has also been revealed^2,3^. Ionospheric perturbations have been linked to electromagnetic pulses during thunderstorms and lightning events^5,6^. A joint possible effect of vertical electrical discharge and atmospheric gravity waves (AGW) on the E-layer has been suggested by Davis and Johnson^7^ as two components of thunderstorm that can influence the background dynamics of the ionosphere. The influence of sprites and streamers from extremely high energy lightning^8^ have been suggested to influence the ionization levels of the ionosphere^9,10^.

A single station and a multi-station study of ionospheric D-layer lightning effect has also been considered by some other investigators^11,12^, and they reported some corresponding perturbations in the D-layer during the lightning event in both studies. The initial study on the influence of thunderstorm on the ionospheric total electron content (TEC) revealed some TEC deviations based on the associated gravity wave effects of the thunderstorms studied^13–15^. However, to the best of our knowledge as at the time of this work, there was no consideration for lightning and thunderstorm effect on measured ionospheric TEC over the latitudes between $\pm 10^\circ$ of either geographic equator or magnetic equator in the previous works, as most of the stations considered in previous investigations are well above $\pm 14^\circ$ which is at about ~1500 *km* beyond the equator. The contribution by Kuo and Lee (2015) is based on modelling of the impact of thunderstorm current on ionospheric current and its resulting plasma perturbations^16^. However, our work is based on direct measurement of TEC above thunderstorm events and their responses, with more focus on the gravity wave effects. Although a recent contribution by Tang *et al*. (2019) focused on the contribution of gravity wave, however, their work only covers the low latitude region from ~14°*N* to 25°*N* close to Hong Kong and its surroundings, which is far greater 1500 *km* from low latitude/equatorial region^17^. It is therefore pertinent to consider investigations around the low/latitude equatorial region, considering the fact that the ionospheric processes, dynamics and responses to external influence vary with proximity to the equator. In this work we have considered station that are located between $\pm 5^\circ$ around the equator and $\pm 5^\circ$ off the deep latitude (see Table 1).

**Table 1:** Selected stations, their coordinates and their GPS data network (Dip latitude to 4 d p (Courtesy Coordinated Community modelling Centre (2017)).

Station Code	Name of Location	Latitude	Longitude	Dip Latitude	GPS Data Network
ACRA	Accra	$5.6041$	$0.1798$	$-3.4736$	AFREF
BJNI	Nikki	$9.9368$	$3.2131$	$-0.9479$	AFREF
BJCO	Cotonu	$6.3703^\circ N$	$2.3912^\circ E$	$-3.0856$	AFREF/IGS
BJKA	Kandi	$11.1305$	$2.9326$	$-0.2341$	AFREF
BJAB	Benin Republic	$6.4200^\circ N$	$2.3500^\circ E$	$-3.0534$	AFREF
BKFP	Birnin-Kebbi	$12.4318^\circ N$	$4.1956^\circ E$	$0.6017$	NIGNET
CGGT/CGGN	Torro	$10.4465^\circ N$	$9.2206^\circ E$	$-0.5634$	NIGNET/IGS
NKLG	Libreville	$0.3962^\circ N$	$9.4673^\circ E$	$-7.9861$	IGS
ULAG	Lagos	$6.5244^\circ N$	$3.3792^\circ E$	$-3.0227$	NIGNET
UNEC	Enugu	$6.4584^\circ N$	$7.5464^\circ E$	$-3.2066$	NIGNET
FUTY	Yola	9.1994	12.4954	$-1.3871$	NIGNET
DAKR	Dakar	$14.7212^\circ N$	$-17.4395^\circ N$	2.2888	IGS

The study of the effect of thunderstorms on the African equatorial sector of the ionosphere is very important but challenging considering the peculiarities of the dynamics of the equatorial ionosphere, as the equator is generally known for a high degree of irregularities and high magnitude TEC gradients most especially at nighttime. These irregularities could be associated with plasma depletion, plasma bubbles from spread F and sporadic E layer disruption or its formation with instabilities during pre-reversal enhancements (PRE) periods and other Rayleigh-Taylor instability based irregularities. Also, phenomena like the complex flow of the plasma such as equatorial electrojets (EEJ) and counter electrojets (CEJs) contribute to the complexity of this region. Another important consideration is that the West-African sector and particularly the Congo sector of the equatorial region accounts for some of the most intense and highest rate of thunderstorms on the globe, and as a result, the Congo basin is seen as one of the lightning chimneys of the globe. Based on these considerations, we hereby focus on the following points of inquiry: The first point of inquiry is on how evident the TEC deviations are, due to thunderstorms at the equatorial regions, considering the high TEC gradients which are peculiar to the equatorial ionosphere especially at night. The second is to examine the variations of these TEC deviations due to thunderstorms, with time, distance, direction and rate of propagation. Third is to compare the nighttime and daytime TEC deviation with more emphasis on the daytime thunderstorm events. Although other studies considered nighttime in other regions, our emphasis in this work is majorly on the daytime ionospheric responses to thunderstorm, as we suspect that the nighttime TEC deviations due to thunderstorm might be negligible around the equatorial region. However, we also considered the nighttime responses for the purpose of comparison and validation.

The TEC response to thunderstorm during other solar events like the solar flare and CMEs were also considered. The mechanisms of the ionospheric responses to thunderstorm under these conditions have been explained in this work. The results obtained from these inquiries could stir up further studies based on the parameters observed, in terms of modeling the ionospheric responses to thunderstorm and also for the improvement of existing models in respect to the degree of accuracy with which they can describe of the dynamics of the equatorial ionosphere.

## Results

**Figure 1:**
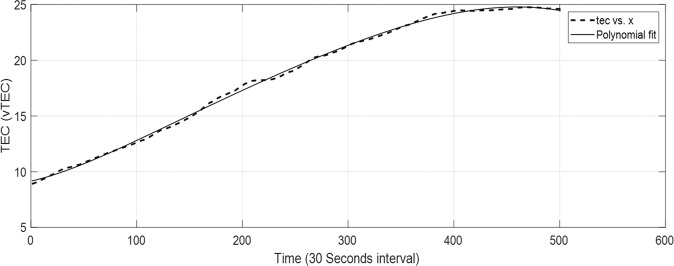
A typical vTEC for a satellite track and its polynomial fit.

The results presented in this work show the TEC deviations for some GPS TEC stations located within the region of the selected thunderstorm events (see Table 1), compared with other stations visible to the same GPS satellites at various distances from the point of thunderstorm event. The TEC deviation was extracted using the method of polynomial filtering. Figure 1 shows a typical vertical TEC (vTEC) for a satellite track and its polynomial fit. The exact time and location of the thunderstorm events presented in this work have been extracted using the World Wide Lightning Location Network (WWLLN) data resources.

### Increased TEC deviation due to thunderstorm and its spatial variation

**Figure 2:**
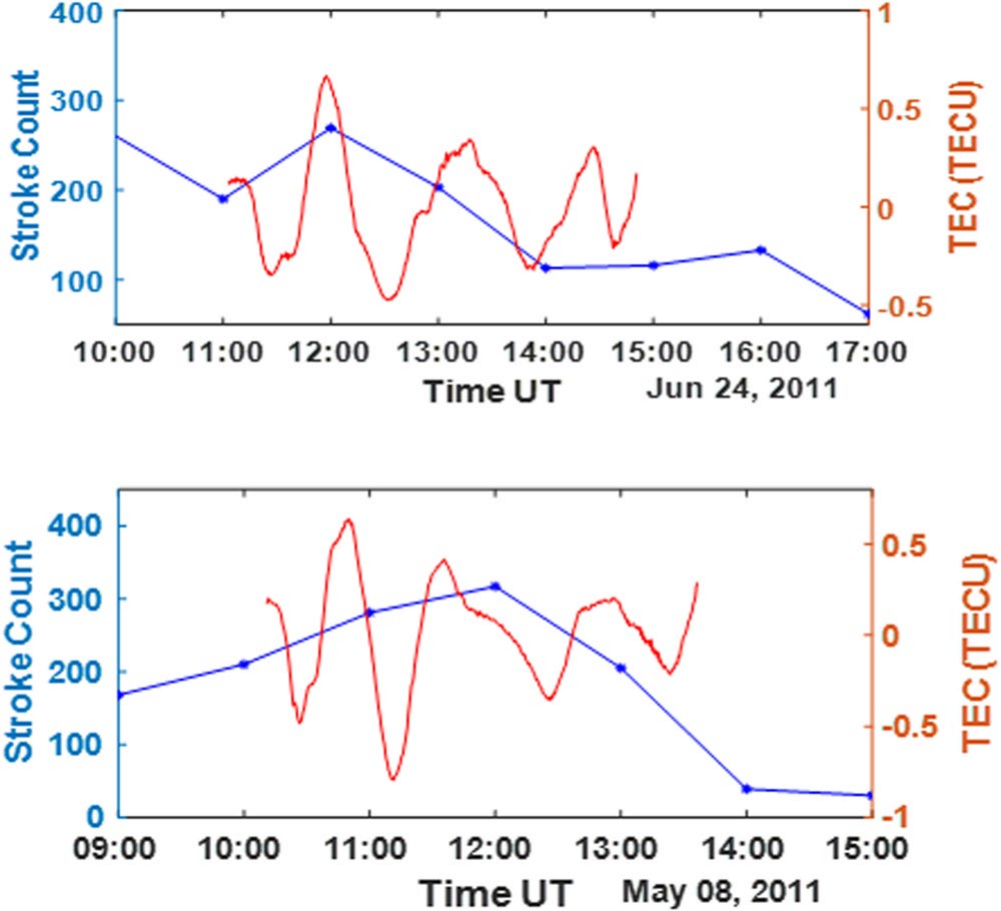
Upper panel: Flash count for June 24 2011 in Lagos with high intensity between 11:00 and 12:00 hours (blue line); Extracted TEC deviation (with gravity wave signature) extracted from PRN 12 track on June 24, 2011 for the ionosphere over Lagos (red line). Lower Panel: Flash count for May 8, 2011 in Lagos with high intensity between 10:00 and 13:00 (blue line); Extracted TEC deviation (with gravity wave signature) extracted from PRN 28 track on May 8, 2011 for the ionosphere over Lagos (red line).

The stroke count and the TEC deviations observed due to the thunderstorm event at Lagos (ULAG) in June 24, 2011 have been presented in Fig. 2 (upper panel) respectively. The thunderstorm-associated lightning occurred actively from around 07:00 UT. The lightning stroke showed a high VLF energy of ~9 *kJ* at 11:06 UT, ~9 *kJ* at 11:32 UT and ~8 *kJ* at 11:38 UT. The return stroke count within this period is greater than 200 strokes per hour for WWLLN and over 1000 stroke counts from 10:00 (see Fig. 2 upper panel). Furthermore it is noteworthy to understand that the WWLLN return stroke usually accounts for only about 20% of the total number of flashes or less^18,19^. The flashes intensified to the highest rate from around 10:00–12:00 UT, with one of the most intense flash episodes between 11:30 and 12:00 hours. The highest TEC deviation magnitude was observed at this same period with highest flash count (see Fig. 2 upper panel (red curve)).

**Figure 3:**
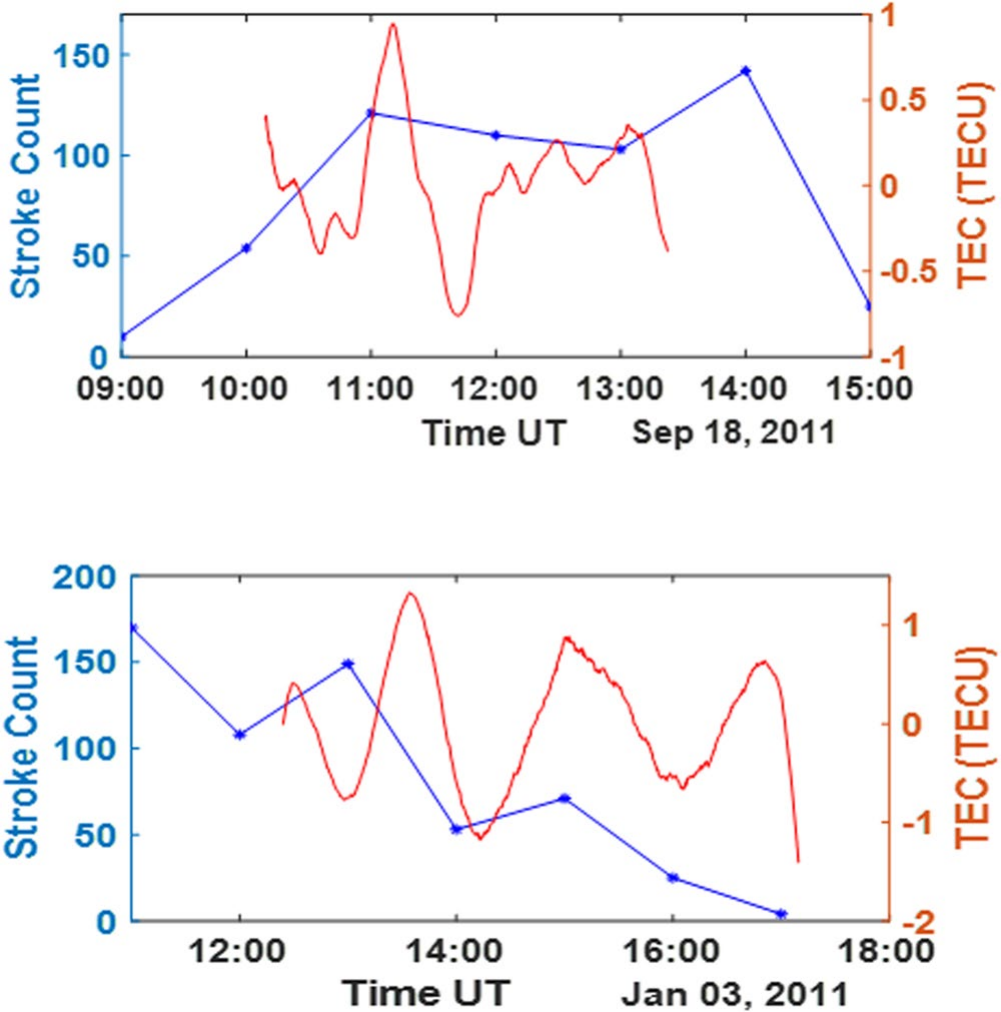
Upper panel: Flash count for September 18, 2011 in Lagos (with high intensity between 11:00 and 13:00 hours)(blue line); Extracted TEC deviation (with gravity wave signature) extracted from PRN 21 track on June 24, 2011 for the ionosphere over Lagos (red line). Lower Panel: Flash count for January 3, 2011 in Libreville (with high intensity between 11:00 and 13:00 hours) (blue line); Extracted TEC deviation (with gravity wave signature) extracted from PRN 20 track on January 3, 2011 for the ionosphere over Libreville (red line).

Similar response to high flash rate have been observed in Lagos on the 8^th^ of May 2011 (see Fig. 2 lower panel) and on the 18^th^ of September 2011 (see Fig.3 upper panel). This can also be attributed to the convective processes, as the thundercloud movement during these events tend to induce gravity waves. The two other cases of high TEC deviation peaks during large thunderstorm, which were observed in Lagos tend to show a similar response to thunderstorm compared with the first case in which there is increase in the peak TEC deviation close to the period of time with highest stroke count. The associated intensified lightning count during the event period which has also been presented in the previous works^20^ accounts for the presence of thunderstorms during that period and the effect of the thunderstorm event within this period was observed in the TEC deviations, as indicated by the wavelike peaks of TEC deviation.

The thunderstorm at Libreville (see Fig. 3 lower panel) between 12:30 and 15:00 hours on January 3, 2011 reveals one of the largest TEC deviations at $\sim \pm 1.35$ TECU observed by PRN 20 satellite (see Fig. 3b lower panel). This value is in the same range as the highest values of TEC deviations presented in previous works^13,15^. The highest VLF energy of the thunderstorm associated lightning recorded by WWLLN is ~16 *kJ*, with a considerable number of return strokes, within about 250 km radius. It clearly revealed the effect of the thunderstorm event, with a rapid increase in TEC deviation at the time of the event as seen in Fig. 3 (lower panel). The wave like perturbation at Libreville and other cases might be due to the effect of gravity waves, which have been suggested by different investigators^15,21,22^.

### TEC deviation propagation and thunderstorm effect

**Figure 4:**
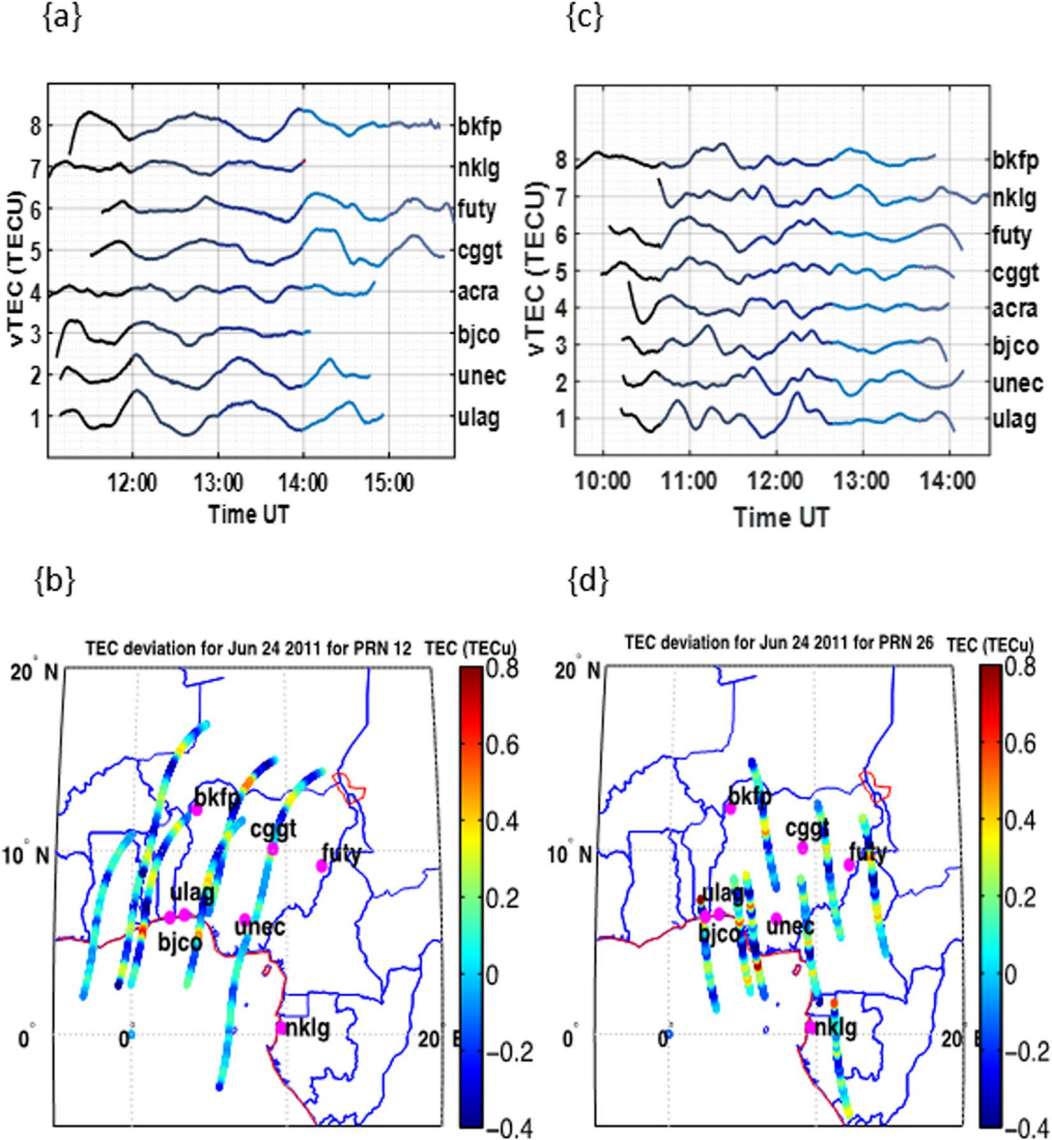
Line plots for TEC deviations during the June 24, 2011thunderstorm event (Top panels) and corresponding TEC deviations along the satellite path for the selected stations (Bottom panels). Figures 4a,b represents the PRN 12 measurements at Lagos for June 24 and while Fig. 4c,d represents PRN 26 measurement at Lagos on the same date.

It was observed that the variations in the highest magnitude of TEC deviations at other stations close to the region of thunderstorm events could be dependent on the direction of the thunderstorm effect. For instance, the maximum TEC deviation due to the thunderstorm event that took place at Lagos on the 24^th^ of January have been found to be propagated in the eastward direction from Lagos (see Fig. 4 upper panel; see also Fig. S3a in the Supplementary). The highest magnitude of TEC deviation measured in Lagos is about $\sim \pm 0.7$ TECU the closest station in the eastward direction is Enugu (UNEC) is $\sim \pm 0.55$ TECU. However, UNEC is not the closest to ULAG station. The closest station to ULAG is the Cotonou station (BJCO) which is in the westward direction. However, the TEC deviation measured at the Enugu (UNEC) station, which is approximately ~450 *km* away from Lagos, appear to be larger compared to the TEC deviation at BJCO. A good number of the other stations in the eastward direction have also been found to have higher magnitude of TEC deviation compared to BJCO. The TEC deviation along the satellite paths (in Fig.4b,d) reveal the deviation of TEC over Lagos the (location of event) and the neighboring stations, showing the magnitude of TEC deviation along the respective GPS satellite paths over each station. The observation made from the TEC deviation along the satellite track reveals that the TEC disturbance propagates northeastwards from Lagos through Enugu all the way to Yola (See Figs. 4b,d).

**Figure 5:**
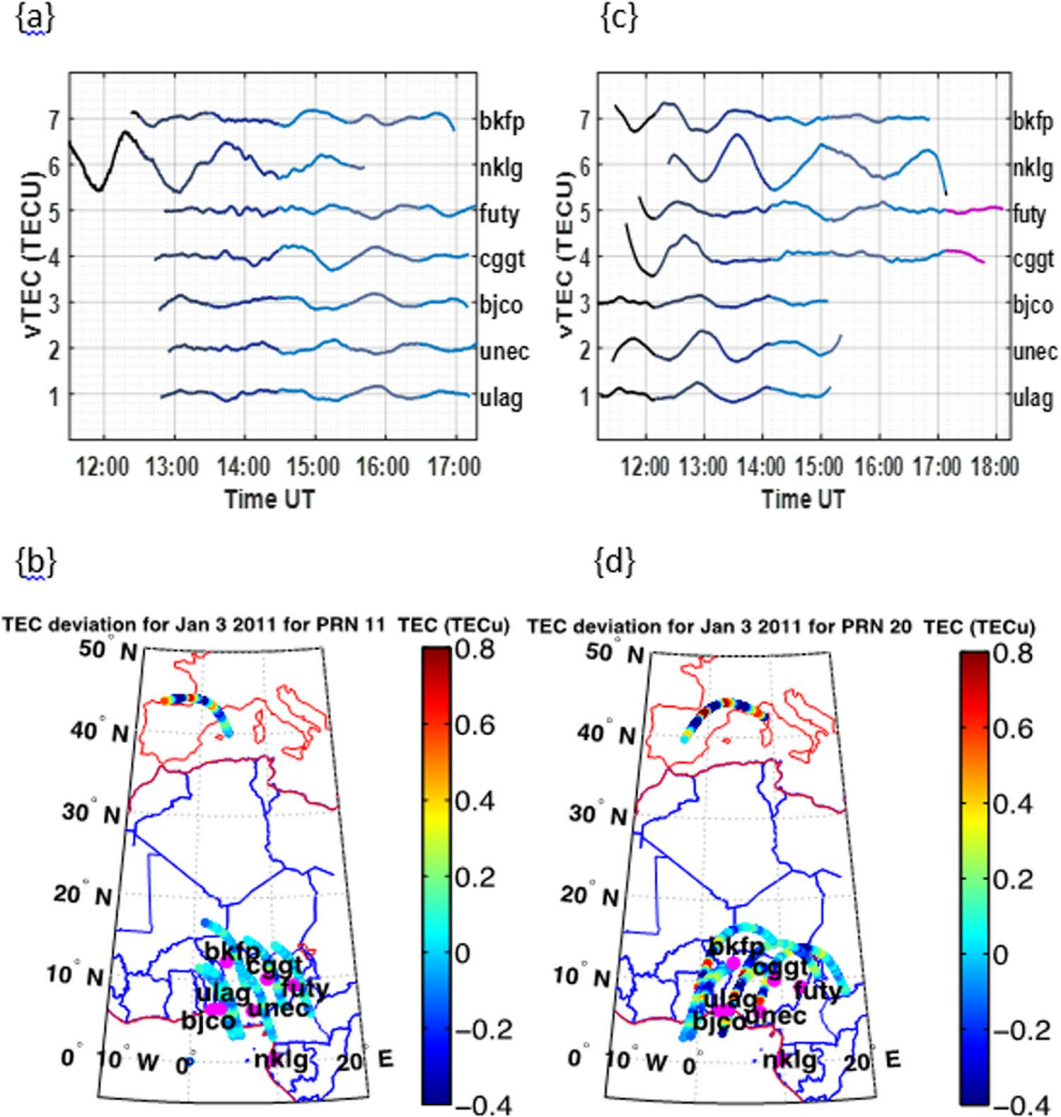
Line plots for TEC deviations during the January 3, 2011thunderstorm event (Top panels) and corresponding TEC deviations along the satellite path for the selected stations (Bottom panels). Figure 5a,b represents the PRN 11 measurements at Libreville for January 3, 2011 and while Fig. 5c,d represents PRN 20 measurement at Libreville on the same date.

The estimated propagation area of the highest TEC deviation from the sky plot (grid estimation from the contour plot) is ~200000 *km*^2^ towards the northeast. The TEC deviations along the satellite path at Libreville on the 3^rd^ of January (see Fig. 5b,d) reveals a north-westward propagation of the thunderstorm effect on the TEC deviation. The thunderstorm associated gravity wave could be responsible for the magnitude of TEC deviation in the same direction, as it reduces with distance. The estimated area of propagation from our observation is $\sim 160000\,k{m}^{2}$ toward the northwest.

In all these cases, it was generally observed that the TEC deviations due to the thunderstorm effect is not totally dependent on the proximity to the point of thunderstorm event, as the direction of propagation is another major factor. Also, that the resulting propagation and spread of the TEC deviation due to the thunderstorm effect is mostly in a specific direction.

### Changes in dynamical variation due to thunderstorm

**Figure 6:**
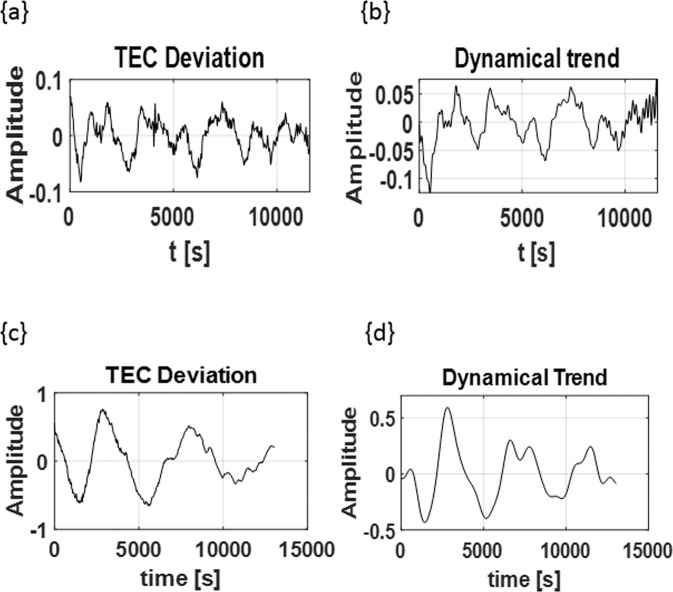
Upper panel: Wave trend extraction using DFT method (**A**) Original TEC deviation signal, (**B**) Extracted wave form for a Day without thunderstorm (PRN 20 track on January 13, 2011 over Lagos). Lower Panel: (A) Original TEC deviation signal, (**B**) Extracted wave form for a day with thunderstorm (PRN 11 track on January 3, 2011 over Libreville) with lowest period of the dominant dynamics oscillating at 0.0002299 Hz (72.5 minutes).

The trend of TEC deviation is usually in an irregular non-sinusoidal form. However during the period of events, the highest magnitudes of TEC deviations due to the thunderstorms appear in smoothened sinusoidal trend (Figs. 2, 3 and 6). The sharp-peaked lower scaled dynamics were observed more when there is no thunderstorm or when the thunderstorm effect is lower (see Fig. 6a). The irregular saw tooth-like transient variations are mainly believed to be the combined transient variation of the ionospheric internal dynamics. The smoothened sinusoid-wavelike dynamics of the TEC deviations during the thunderstorm events (Fig. 6c) may be due to the AGW modification of the internal dynamics at the peak of the thunderstorm. A frequency spectrum analysis based on the method of Digital Fourier Transform (DFT) was used to analyze the TEC deviations and the extracted dominant waveforms from this process were mostly found to oscillate between the range of ~16 *min* and ~76 *min* during large scale thunderstorm events (see Fig. 6b,d).

### The effect of thunderstorm on solar activity modified ionosphere

**Figure 7:**
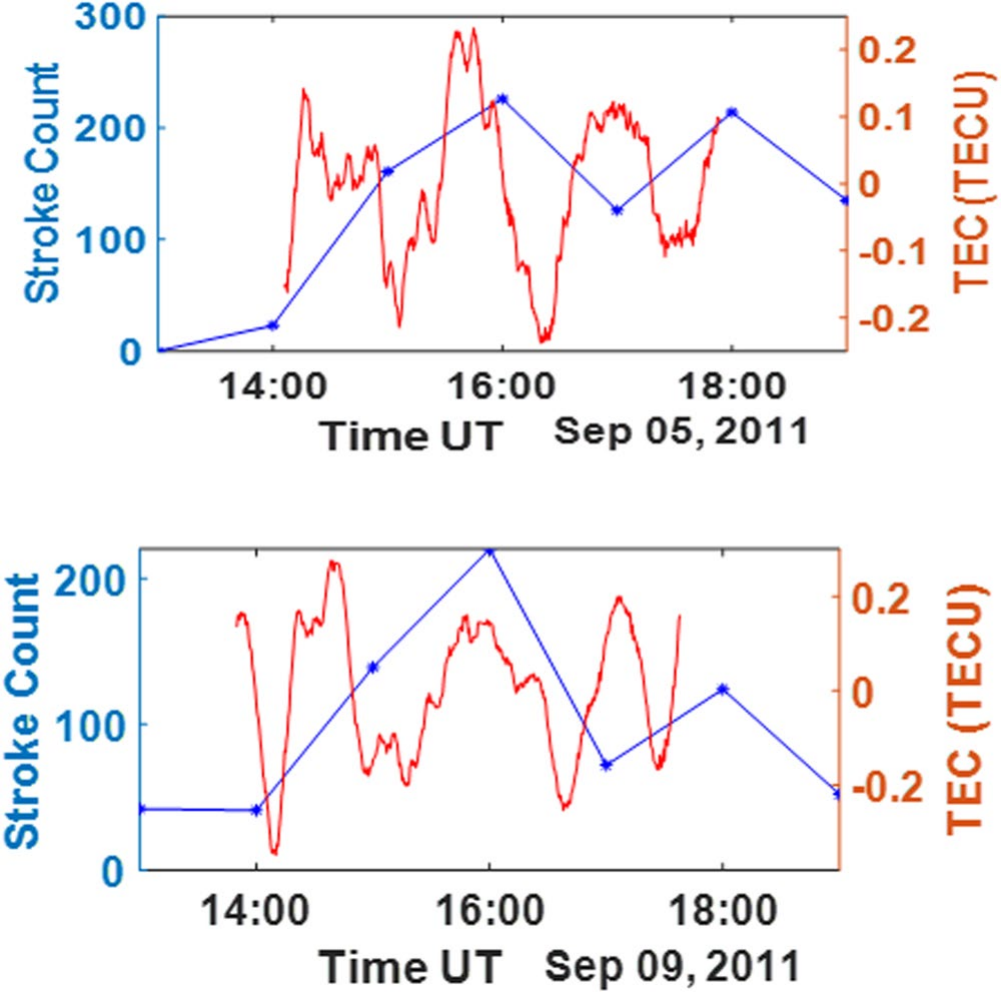
Lower TEC deviations due to solar flare effect. Upper panel: Stroke count for Sep 5, 2011 lightning and thunderstorm at Lagos (blue line); the corresponding low magnitude TEC deviation from PRN 22 (red line). Lower Panel: Stroke count for September 9, 2011 lightning and thunderstorm at Lagos (blue line); the corresponding low magnitude TEC deviations PRN 22(red line).

**Figure 8:**
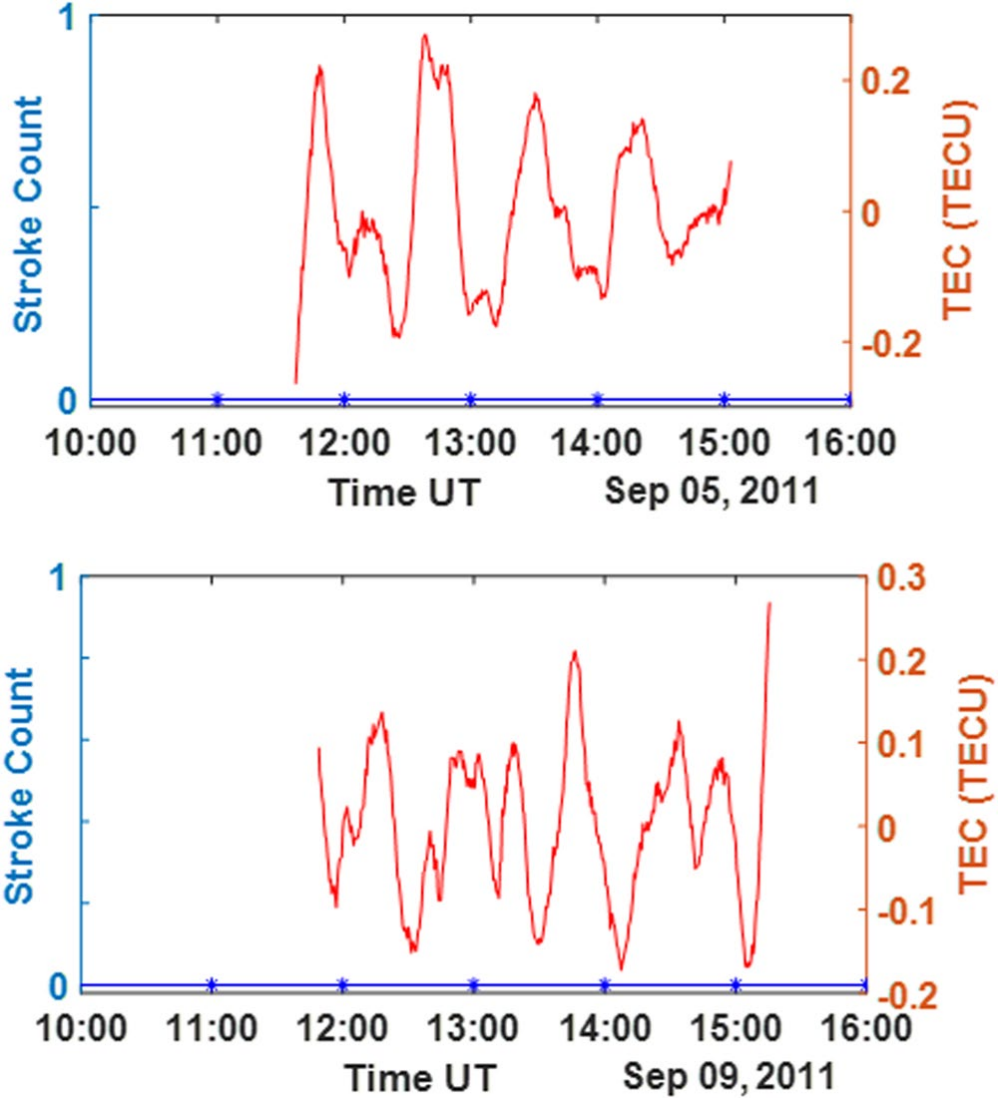
Lower TEC deviations due to solar flare effect without thunderstorm. Upper panel: Stroke count for Sep 5, 2011 lightning and thunderstorm at Dakar (blue line) indicating lack of thunderstorm; the corresponding low magnitude TEC deviation from PRN 21 (red line). Lower Panel: Stroke count for September 9, 2011 lightning and thunderstorm at Dakar (blue line) indicating lack of thunderstorm; the corresponding low magnitude TEC deviations PRN 21 (red line).

The thunderstorm effect on the ionosphere was evaluated for a number of solar events, which includes a geomagnetic storm, solar flares and a corolla mass ejection (CME). Although the effect of geomagnetic storm has been previously considered, another case of geomagnetic storm was considered in this work for comparison with a previous work^13^. The results obtained for the thunderstorm on 5^th^ of August (a geomagnetic storm day) have been found to be similar to the results from previous work^13^. The second consideration in this work for solar events is the case of solar flare conditioned ionosphere (see Fig. 7). A series of lower values of TEC deviations were recorded during the thunderstorm events on the 5^th^, 8^th^ and 9^th^ of September 2011 in spite of the lightning intensity, whereas, larger values of TEC deviation have been associated with similar amounts of flash intensity or even less in the absence of large geomagnetic events. The moderate thunderstorm effect on TEC recorded at this period in Lagos might be due to the series of solar events from 5^th^ to 9^th^ of September. The earth was bombarded by a series of class X and class M flares from the geo-effective filaments at sunspot 1283 on these dates together with a CME impact on the 9^th^ of September^23–25^. The waves of ionization due the solar flare might have resulted in an increased rate of ionization and recombination processes in the ionosphere, thereby modifying the effect of thunderstorm^26^. The impact of the solar flares and the CME might have modified the effect of the thunderstorm. A similar response can be reported for the solar events that took place due to active solar region 1302 from September 24 to September 27, 2011 (with solar flare on September 24 and also with CME impact and severe geomagnetic storm on September 26) which might have resulted in the lower values of TEC deviations (see Supplementary Table 3). A remote station with the absence of thunderstorm during solar flare was chosen to compare its TEC deviation waveform with TEC deviations from Lagos, a station with the two events. The TEC deviation measured in Dakar during solar flare in the absence of thunderstorm shows that the trend is non sinusoidal and similar to the signal in a station with both solar flare and thunderstorm (see Fig. 8). This indicates that the influence of solar flare is more dominant compared to the effect of thunderstorm on the ionosphere.

### Insignificant effect of the nighttime thunderstorm on the equatorial TEC

**Figure 9:**
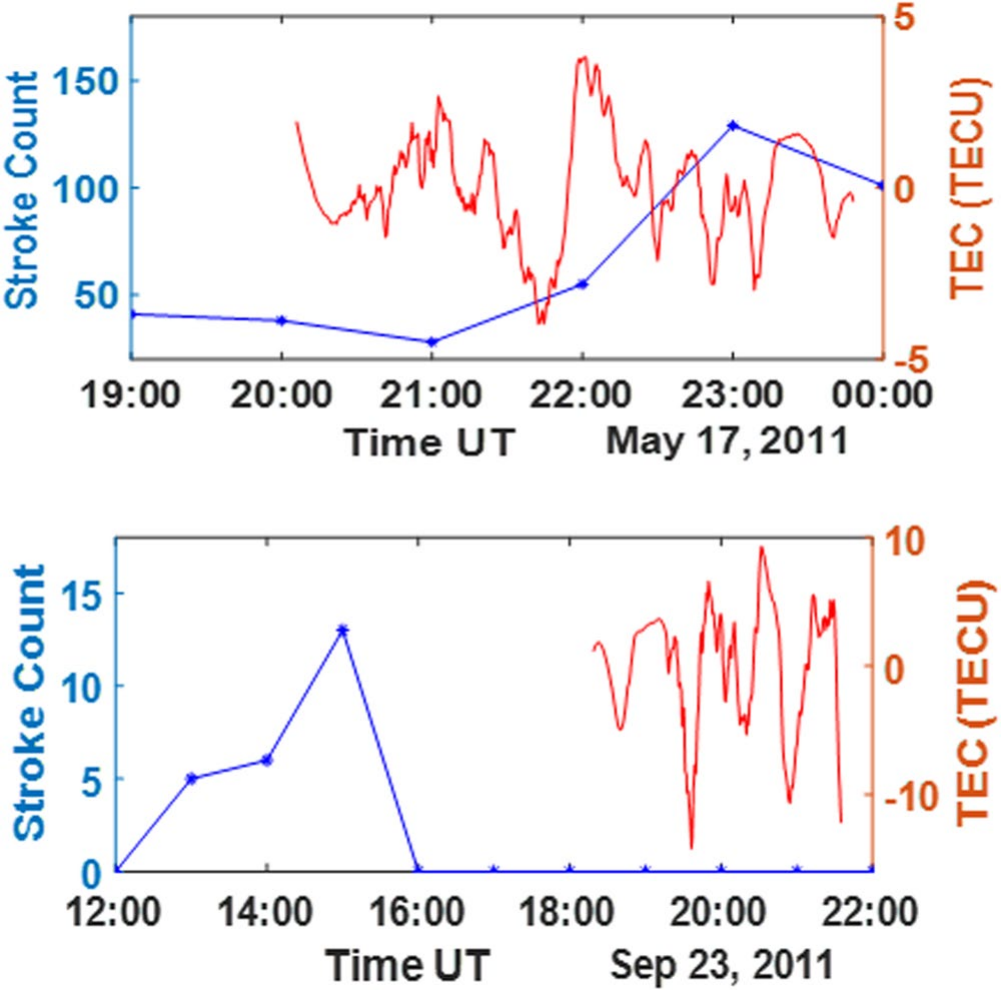
Upper panel: Stroke count for May 17, 2011 indication a presence of lightning and thunderstorm event (blue line); the corresponding post sunset TEC deviation during lightning flash without a visible gravity wave signature (red line). Lower Panel: Stroke count for September 23, 2011 indication absence of flash count at post sunset (blue line); the corresponding post sunset TEC, which is enhanced without Thunderstorm (red line).

To further validate the effect of nighttime thunderstorm on the equatorial ionosphere, we selected nighttime events for months characterized by frequent lightning in Lagos, Nigeria (see Table 2 and Supplementary). The results from this investigation revealed that the effect of thunderstorm on the post-midnight equatorial ionosphere (between 1:00 and 5:00 hours) is mostly negligible. However, large scale enhancements and depletions in TEC with sharp transient peaks were observed within the post sunset period between 19:00 and 24:00 hours with or without thunderstorms. As a consequence of the observed high magnitude sharp peaks, the gravity wave dynamics seems invisible during thunderstorms occurrences within these periods. The nighttime deviations without thunderstorm observed in this work ranges between 1 TECU and 14 TECUs (see Fig. 9 lower panel). The TEC deviation extracted for nighttime are mostly found to possess a dominant irregular dynamics that cannot be attributed to gravity wave (see Fig. 9 upper and lower panel). Also, contrary to the day time results, the nighttime magnitudes of TEC deviation values are inconsistent with the location of thunderstorm event (see Table 2). The inconsistency in values may be due to local night time dynamics.

**Table 2:** The approximate absolute peak TEC deviations showing nighttime thunderstorm proximity independence (values measured at active thunderstorm locations marked with*; ND represents no data).

Date	PRN	ULAG	UNEC	NKLG	FUTY	BJCO	BKFP	ACRA	CGGT
Oct 8, 2011	5	0.539*	0.626	0.105	0.529	0.927	0.529	0.175	ND
Sep 27, 2011	12	0.169*	0.457	0.192	0.630	0.147	0.916	0.118	ND
Aug 20, 2011	19	0.520	0.746*	0.614	1.009	ND	0.939	2.052	1.421
Oct 29, 2011	5	0.623	1.07	0.548*	1.00	ND	0.914	0.795	2.038
Sep 23, 2011	23	1.115	1.200*	14.21	1.444	1.212	ND	3.271	1.8

The nighttime dynamics of the equatorial ionosphere is usually characterized by large and sporadic TEC gradients, compared to other regions like the mid-latitudes^27^. The effect of frequently observed high magnitude nighttime thunderstorm on the equatorial ionospheric TEC deviations have been observed to be generally invisible in the ionospheric dynamics, due to post sunset sporadic variations in the equatorial ionosphere, especially between 19:00 and 24:00 hours. This period is characterized by large TEC deviation or depletions that renders the effect of thunderstorm on TEC negligible^27–31^. For this reason the daytime events were mainly considered in this work while the nighttime events were only selected in this inquiry for the purpose of validation.

## Discussion

The purpose of this work is to evaluate and highlight the impact of thunderstorms on the equatorial ionosphere particularly along the West Africa-Congo sector of the equatorial region. Also, to compare the characteristic dynamical response of the region with other regions. In this section, we discuss the significance of the findings from our result with reference to established knowledge of the equatorial ionosphere. First, it is important to compare the similarities and the differences in the result we obtained to the results previously obtained from the mid-latitude and other regions^13,15^. This work further reaffirms from previous works that the higher amplitude wavelike TEC deviation might have resulted from the AGWs associated with the thunderstorms. Similar to previous works^13^, the results obtained reveal that there is a higher level of TEC deviation at the point of thunderstorm event with deviation patterns that can be attributed to atmospheric gravity waves, as the TEC deviation pattern reveals smooth peaks of deviations around the time of event. This trend appears to be mainly evident within the region around the point of the thunderstorm events most especially at the point of the event, with change in dynamics afterwards, as other variations which are probably due to the internal dynamics re-emerge (see Figs. 2 and 6). A possible explanation of this change in characteristics is the change in dynamics due to the suppressed dynamics of the system during the thunderstorm. The internal dynamics of a system like the ionosphere can be easily modified due to external influence such as thunderstorms (see Fig. 6b upper panel).

However in comparison to previous work^13^, even though the TEC enhancements due to thunderstorms reduces with distance from the point of the thunderstorm, we have been able to show from our result that these variations may not only depend on the proximity of the other stations to the station at the point of the thunderstorm occurrence, but might be partly dependent on both direction and proximity to the station. A typical example is the thunderstorm occurrence in Lagos on June 24, 2011. Our main inference from this observation is that propagation of the thunderstorm effect revealed by the TEC deviations might be due to the direction of the AGW effects associated with the thunderstorm.

Furthermore the propagation of the TEC deviation due to the thunderstorm effects were mostly in a specific lateral direction contrary to the assumption of the possibility that its lateral propagation could be approximately radial. This might be due to the transverse lateral propagation of the gravity wave effect. As the thunderstorm effect on the TEC spreads out from a source (the point of thunderstorm effect) in a specific direction to other stations, it fades out with distance (see Fig. 4(b,d); see also Supplementary Fig. S3). The spatial distribution variation of the thunderstorm effect of TEC requires further investigation in future with a well distributed GPS TEC measuring station.

Previous works have suggested that AGWs having their periods within the range of 10–80 min and horizontal wavelengths between 100 km and 1200 km can penetrate through the D and E region to the bottom side F region ionosphere from their source in the troposphere^21,22^, suggesting both lateral and horizontal propagation and a wide range of possible speed of propagation. The periodic deviation of TEC due to the thunderstorm effect for most of the observations in this work are mostly found to be within the periodic range of about 16–76 *min*.

Another noticeable observation is that the effect of the solar flare event that occurred the same days as the thunderstorms could have resulted in high rates of ionization and recombination of ions in the ionosphere and hence a stronger influence on TEC variation compared to the effect of the thunderstorm. Solar flare has been previously reported to greatly influence the internal dynamics of the ionosphere due to the effect of its associated high energy radiation^26,32,33^. The implication of this observation is that even though the resulting VLF far field energy of the thunderstorm associated lightning and the number of strokes can determine the magnitude and the possible impact of the thunderstorm on the ionosphere, this far field energy may not be linearly related to the observed TEC deviations due to other possible factors like solar flare. The response of the solar flare modified ionosphere to thunderstorm might be quite unnoticeable.

Contrary to previous works, the gravity wave dynamics and the spatial TEC deviations due to the nighttime thunderstorm events were found to be untraceable in all the considered cases. The nighttime results observed in this work, reveal very large and sharp/transient TEC deviations (see Fig. 9). These sporadic high magnitude nighttime variations can be generally attributed to the enhanced equatorial nighttime TEC gradients^27–31^. The wavelike dynamics of the TEC deviation due to thunderstorm is probably inhibited due to the extremely large nighttime TEC gradients at the equator, as the thunderstorm-associated gravity wave dynamics seem untraceable in most cases, especially 19:00 and 24:00 hours LT. The nighttime TEC gradients can be up to 10 TECUs or even much more in some cases^27^. Also, the proximity independence of TEC gradient magnitude during thunderstorm at night (see Table 2) can be attributed to the characteristic high magnitude nighttime TEC gradients, which might be higher as a result of more prevalent equatorial irregularities. In this work such high magnitude TEC gradients were observed at night during thunderstorm and in the absence of thunderstorm (see Fig. 9b upper and lower panel). Hence, the thunderstorm associated gravity dynamics and the spatial variation of the TEC deviation due to thunderstorm seem undetectable at nighttime, close to the equator.

**Figure 10:**
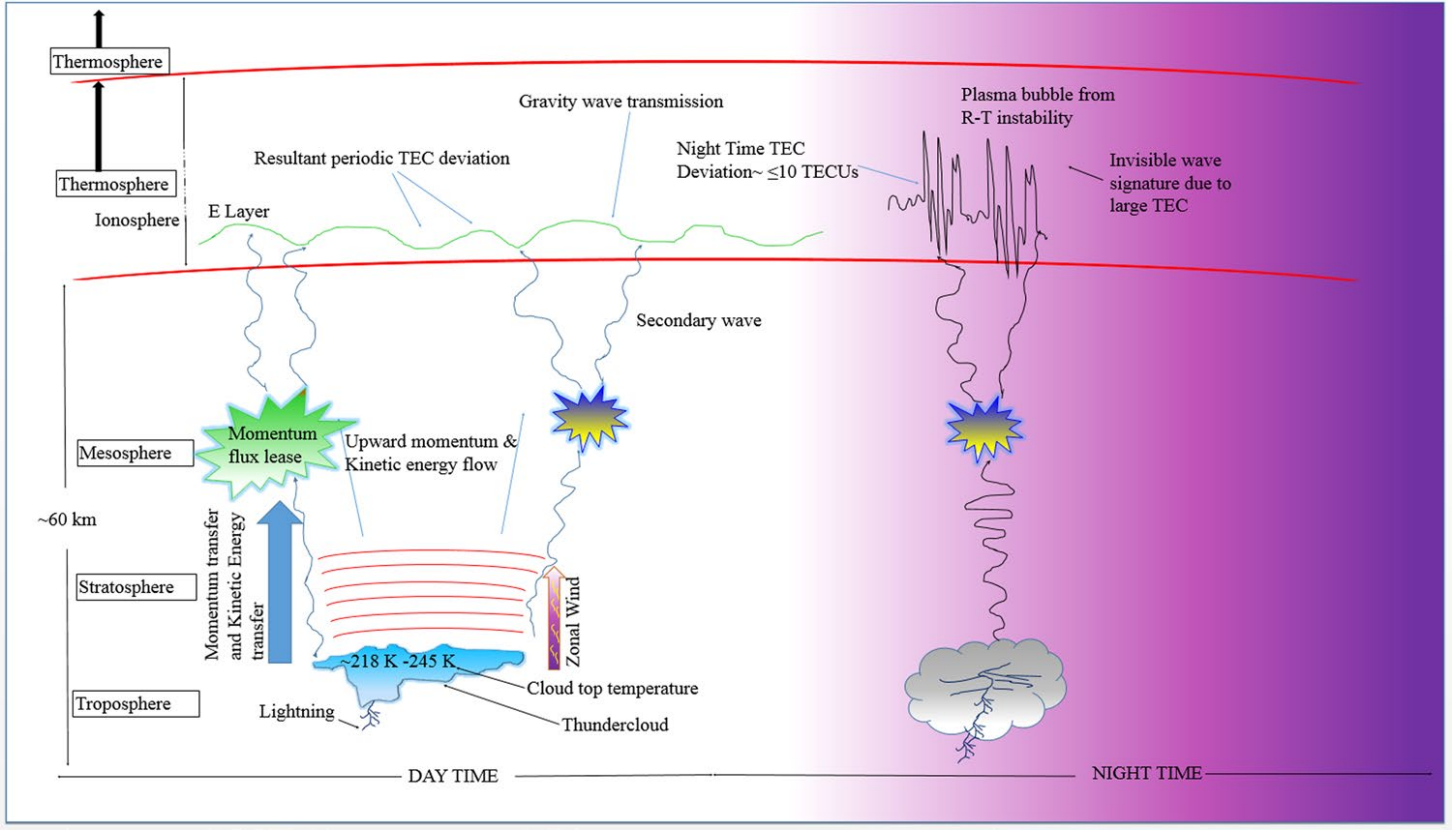
An illustration of the atmospheric wave dynamics from convective processes and ionospheric responses.

Considering the processes discussed in this work, we present our general perspective as follows (see Fig. 10): During convective processes, the thunderstorm produces upward gravity wave effect, which results in the upward transfer of kinetic energy and momentum between the layers of the atmosphere. The wave transfer between the atmospheric layers results in changes in amplitude due upward density gradients. The resulting change in amplitude around the mesosphere could sometimes lead to criticality of the wave such that, the wave breaks and produces secondary waves as it releases momentum fluxes^34^. In this process gravity waves can be transported up to Ionospheric heights leading to perturbations in the ionosphere. The gravity wave impact produces wavelike TEC deviations (which might vary from 0.5 to 1.5 TECUs) during the daytime (See Illustration in Fig. 10). However the nighttime gravity wave signatures from thunderstorms are mostly invisible at night around the equator. The factors responsible for this night time phenomenon includes the Rayleigh-Taylor (R-T) instability, which occurs around the equatorial region. The R-T instability can be seeded due to combined effects of the gravity wave and the eastward electric field in the presence of nighttime equatorial ionospheric density gradient. The R-T instability will consequently produce the EPBs/ESF^35,36^. This could have resulted in inhibition of the wavelike TEC signatures in the computed TEC deviation due to the resulting high background TEC night time gradients (sometimes up to 10 TECUs). Hence, the night time thunderstorm effect becomes negligible under such circumstances.

Also deduced from our observation is that for all the cases considered, the highest number of events and the largest TEC deviations due to thunderstorm were found at Libreville compared to other regions in West Africa considered. This might be due to the fact that Libreville is located within the Congo basin, as extremely high lightning flash rate has been associated with this region^37^.

It is also noteworthy that results by some authors have revealed the possible effects of other factors that can lead to ionospheric perturbations during the thunderstorm and lightning events. For example it has been suggested that the possible vertical transportation of electrostatic discharge might induce perturbations in the ionosphere apart from the effect of gravity waves^7,11^. Also the effect of transient luminous events (such as sprites and halos), which could result in ionization enhancement from the thunderstorm and lightning events has been suggested to induce fluctuations in the D-region of the ionosphere^10^. These results are possible contributions to the thunderstorm-ionospheric dynamical effects. However, the most explainable mechanism based on our findings in this work is the effect of the thunderstorm associated AGWs on the ionosphere during the thunderstorm events.

## Outlook

The deviations of TEC due to thunderstorm effect have been studied in the equatorial region to examine its mechanism of variation and propagation. We examined the direction of the thunderstorm, the spatial spread and speed of propagation of the TEC deviations, resulting from thunderstorm effect and other parameters. A number of inferences were drawn as follows:

i. The daytime equatorial ionospheric revealed significant TEC perturbation, as the internal dynamics of ionosphere is modified by the thunderstorm associated gravity wave.
ii. The gravity wave signature of the thunderstorm effect appear to be invisible at night due to background large scale TEC irregularities.
iii. The spatial variation of TEC deviation is dependent on both proximity and direction of propagation. The TEC perturbation due to thunderstorm is usually propagated in a specific direction from the point of event.
iv. During intense solar activities, the thunderstorm associated gravity wave signature on the solar activity modified ionosphere is usually suppressed in amplitude, as it also appears to be irregular and non-sinusoidal in trend.

Considering the results obtained so far we further affirm that even though the effect of equatorial ionospheric TEC to thunderstorms have been found to be visible, mainly during the day, it is of utmost importance to further study these effects for other equatorial regions of the globe for better knowledge and understanding of the equatorial ionospheric responses to thunderstorm. This can help produce better information for the modeling of ionospheric responses to tropospheric events in future, and to improve existing models to better describe the behavior of equatorial ionosphere.

## Data and Methods

### Data and thunderstorm case selection criteria

In this work, fluctuations in the ionospheric dynamics were examined by extracting the deviations in total electron content (TEC) from satellites visible at the period of thunderstorm events. The TEC data used were obtained from NIGNET, AFREF and IGS data archives. The selected stations, their coordinates and Global Positioning System (GPS) data networks are listed in Table 1. These stations have been selected along the West Africa to Congo basin sector of the equatorial/low latitude region, considering the fact that there are highly frequent thunderstorm events within this region, which produce very frequent lightning activities. The choice of stations is based on three criteria. First, the stations were selected based on their dip latitude, and it is selected between 0° and 8°*N* so as to cover the rainforest region of the West Africa and the Congo basin close to the dip equator. Second, GPS receiver stations along the West African rainforest region and the Congo basin between 0° and 10° longitude were selected, to ensure simultaneous coverage of the same GPS satellite over the selected stations. The third criterion is the availability of TEC data during the observed periods of events. The TEC data from the archives were obtained in Receiver Independent Exchange (RINEX) format data. They were converted to more readable format to obtain TEC through arc by arc method^38,39^. The computation TEC has been carried out using the ionospheric height of 200 km, rather than the regular Ionospheric pierce point (IPP) of 350 km, mostly considered. This is to properly capture the possible tropospheric effects on lower portion of the ionosphere without including the bulk TEC^13^.

The carrier phase measurements were used for the estimation of slant TEC (sTEC), to avoid the noise associated to code measurements following Ciraolo *et al*.^38,39^. However, the unknown ambiguity after each cycle slip or receiver’s loss of lock were determined using least square method to filter and eliminate the biases. In addition, satellites with 30 degree and above elevation in the neighbourhood of the stations location were used in the estimation, to minimize the error in the estimation. It also assists in excluding satellites with locations further away from the coordinate of the selected stations. The satellites and receivers biases were obtained from IGS website. These biases were removed from the TEC estimation to minimize pseudo-range errors. The vertical TEC was obtained by multiplying the slant TEC by the mapping function:

1 $$vTEC=sTEC\times MF$$

2 $${\rm{When}}\,MF=\,\cos (\chi )$$

MF is the mapping function, χ is 90° minus elevation angle $(\varphi )$ of the satellite signal raypath

3 $$\chi =90-\varphi $$

The thunderstorm event periods were selected based on the associated lightning. The lightning days were selected from months characterized by thunderstorm and intense lightning events. The lightning time, event location and the VLF far field energy data were extracted using the World-Wide Lightning Location Network (WWLLN) data resources. With reference to WWLLN data sets, the lightning events within 250 km radius around the GPS TEC receiver stations were selected. The choice of the lightning was based on three criteria. The first is based on the magnitude of the far field VLF energy, and periods with VLF energy magnitudes greater than 10^3^ *J* were selected. The second is that the daytime thunderstorm events between 09:00 hours LT and 17:00 hours LT were considered. This is to avoid the effect of high magnitude post sunset/nighttime ionospheric irregularities commonly observed within the equatorial region^27–30^. However nighttime events between 00:00 and 06:00 hours LT were selected for further comparison. The third criterion is based on the solar activity on the day of the thunderstorm event. To avoid possibilities of high TEC gradient enhancements and depletions due to geomagnetic storm effects, days with the 24 hour maximum *K*_*p*_ index less than 4, and 24 hour minimum D_st_ index greater than −50 were selected in this work. Also based on solar activities, we considered 2 solar flare event day, to observe the effect of the thunderstorm during solar flare event.

### Analysis

The TEC data obtained were analyzed based on the satellite visibility at the point of thunderstorm event. The GPS satellites’ Pseudo Random Noise (PRN) has been used for identification of the visible GPS satellites. The TEC deviations during the thunderstorm were extracted from the computed TEC obtained from the visible PRN with highest angle of elevations at the time of event. Angles of elevation between 65° and 80° were mostly considered in this work. The method of polynomial filtering was used for the extraction of TEC deviations. A polynomial of order 6 was fit to the TEC to extract the disturbed TEC from the measured TEC for the period of the selected events^13,40^. The extraction of the TEC deviation using the polynomial of order six is as follows:

Given a time series *X*(*t*) function from measured series ${x}_{1},{x}_{2},{x}_{3},\,\ldots \ldots ,{x}_{i}$, we can obtain a polynomial

4 $${P}_{n}(X)={q}_{1}({X}^{n})+{q}_{2}({X}^{n-1})+\ldots \ldots +{q}_{n}(X)+{q}_{n+1}$$

where *q*_*n*_ are quantities derived based on *P*. The TEC deviation *T*_*dev*_ is given as:

5 $${T}_{dev}=X(t)-{P}_{6}(X)$$

The extracted TEC deviations were compared between stations based on proximity to the point of thunderstorm events. Also, the extracted TEC variations were further analyzed to obtain the spatial and time variation using the method of sky plot and extrapolated contour. A Digital Fourier Transform DFT based spectrum analysis was carried out on the extracted TEC deviation to reveal the type of perturbations found during thunderstorms.

## Supplementary information

Supplementary information.
