# Supplementary material for: Significant Day-time Ionospheric Perturbation by Thunderstorms along the West African and Congo Sector of Equatorial Region
Source: Sci Rep. 2020 May 21;10:8466. doi: 10.1038/s41598-020-65315-3 (PMC7242353; doi:10.1038/s41598-020-65315-3)
Supplement: Supplementary file 1 — Supplementary information. [file 41598_2020_65315_MOESM1_ESM.docx]

Supplementary Information

Article in Scientific Reports

**Significant Day-time Ionospheric Perturbation by Thunderstorms along the West African and Congo Sector of Equatorial Region**

Ogunsua B. O.^1, 2^ Srivastava A.^1^ Bian J.^1,3^ Qie X. ^1,3^ Wang D.^1^ Jiang R.^1^ and Yang J.^1^

^1^Key Laboratory for middle Atmosphere and Global Environment Observation (LAGEO),

Institute of Atmospheric Physics (IAP), Chinese Academy of Science, Beijing, China.

^2^Department of Physics, Federal University of Technology, Akure, Nigeria.

^3^College of Earth and Planetary Sciences, University of Chinese Academy of Sciences, Beijing, China

_Corresponding Authors: Qie X.: qiex@mail.iap.ac.cn and Ogunsua B. O.: bogunsua@gmail.com_

This supplementary material show additional content to support the results provided in the main text. The contents of the given additional materials (text, data and figures) include different related cases that could not be added to the main text.

**Supplementary Results**

**Variations in TEC Deviations in Libreville: Other Cases**

The direction of propagation of the TEC deviations for the 10^th^ October 2011 event between 11:00 and 12:00 in Libreville is similar to that of 3^rd^ of January, from the same station (see Fig. S1 Panel B and D). The magnitude of the TEC deviation is larger at Libreville compared to the other neighbouring locations. As estimated, the observed propagation is $\sim180000 {km}^{2}$ northwest as observed from the sky plots for PRN 18. Considering the magnitude of the highest far field VLF energy estimated by WWLLN which is $\sim18 kJ$ within about100 km radius and $\sim30 kJ$ within 200 km radius comparison with the vertical TEC deviation, our deduction from this is that even though the effect of the far field VLF energy from WWLLN data may not be highly significant on the vertical TEC deviation, a much higher effect on the lateral spread might be possible. Although very high lightning energy may not be the only factor attributed to size of the thunderstorm it is considered as one of the indicators of large scale thunderstorms. Our inference suggests that the size of the thunderstorm is responsible for the lateral spread of the TEC deviation in this case. Considering the direction and spread of the thunderstorm propagation for the two events at Libreville (on the third of January and on the 10^th^ of October), the total spread limit towards the east cannot be ascertained in both cases at this time due to unavailability of accessible GPS receiver stations towards the northeast of Libreville. However, despite this limitation we were able to see that a highly significant part of the propagation effect of the thunderstorm on TEC towards the north-west in both cases.

The 5^th^ of January events between 11:00 and 14:00 hours period of the day revealed the lowest amount of energy at $\sim7 kJ$ and a smaller amount of strikes within the range of $\sim100 km$ radius of the coverage area, compared to the other cases considered. It can be clearly observed (see Fig S2) that the values of TEC deviation were far lower compared to the values recorded in other cases. The lower values of TEC deviation in this case can be attributed to the possibly lower intensity thunderstorm, as inferred from the associated lightening recorded by WWLLN.

In all these cases the propagation of the TEC fluctuations due to the thunderstorm effect appear to be mostly in specific directions in virtually all the cases, however the high magnitude thunderstorm in August 5, 2011 at Enugu appears to reveal a radial propagation. With a larger portion of spread toward the north east. The high degree of lateral spread may not only be due to the magnitude of the high magnitude of thunderstorm, as the associated far field VLF energy measured by WWLLN (values measure at 10:30 UT and 11:30 UT were $>16 kJ$ ). This because the added effect of the geomagnetic storm on the same day, which might have suppressed the value of TEC deviations due to the thunderstorm.

**TEC disturbance propagation from the contour perspective**

Considering the propagation of the TEC disturbance during thunderstorm, four additional diagram are shown in figure S3. For the purpose of illustration, the contour is made by interpolation of the absolute values of the TEC deviation maxima, using the kriging method. The contour plots corroborate the TEC propagation directions revealed by the plot of TEC deviations along the satellite path as seen in Figs.4 and 5 in the main paper, as well as Fig S1 and S2.

**Signatures of TEC deviation in the absence of Thunderstorm**

The Lagos station was chosen for the examination of days without thunderstorm. The days without thunderstorm in Lagos were mostly found in January according to the WWLLN data. Although some part of December also February also lack thunderstorm and rainfall. However, the main period of the break season was seen in January. Fig. S6-S8 shows the time series plot for some days without thunderstorms in Lagos for January 2011. The results show that the trends of the TEC deviations obtained for this period tend to possess irregular saw-tooth wavelike non-sinusoidal patterns. Also, the magnitudes TEC deviation in the absence of Thunderstorm is usually very low mostly below 0.2 TECUs, which is exceeding lower than the values obtained during Thunderstorm.


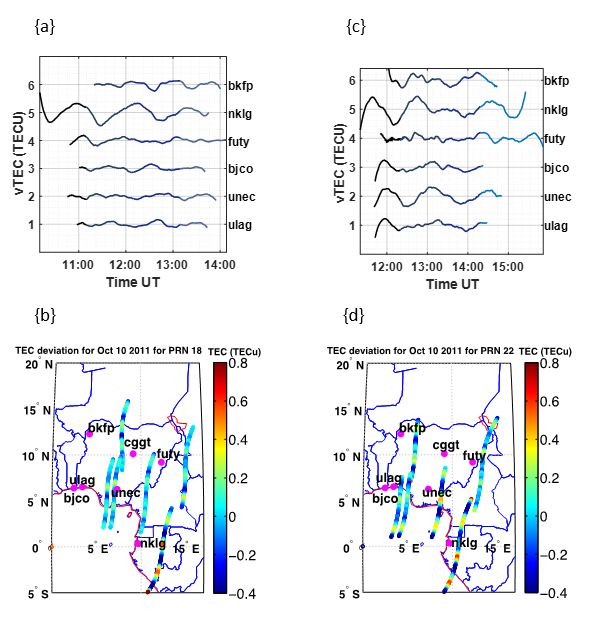


Fig. S1 Line plots for TEC deviations during thunderstorm events (Top panels) and corresponding TEC deviation along the Satellite path for the selected stations (Bottom panels). a and b, for PRN 18 measurement of the October 10, 2011 thunderstorm event, observed at Libreville. c and d for PRN 22 measurement of the October 10, 2011 thunderstorm event, observed at Libreville.


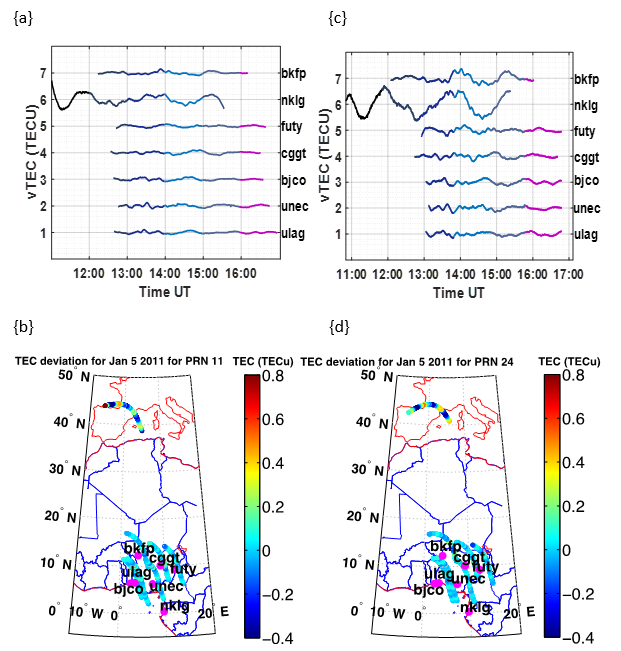


Fig. S2 Line plots for TEC deviations during thunderstorm events (Top panels) and corresponding TEC deviation along the Satellite path for the selected stations (Bottom panels). Fig. a and b, is for PRN 11 measurement of the January 5, 2011 thunderstorm event, observed at Libreville. Fig. c and d is for PRN 24 measurement of the January 5, 2011 thunderstorm event, observed at Libreville.


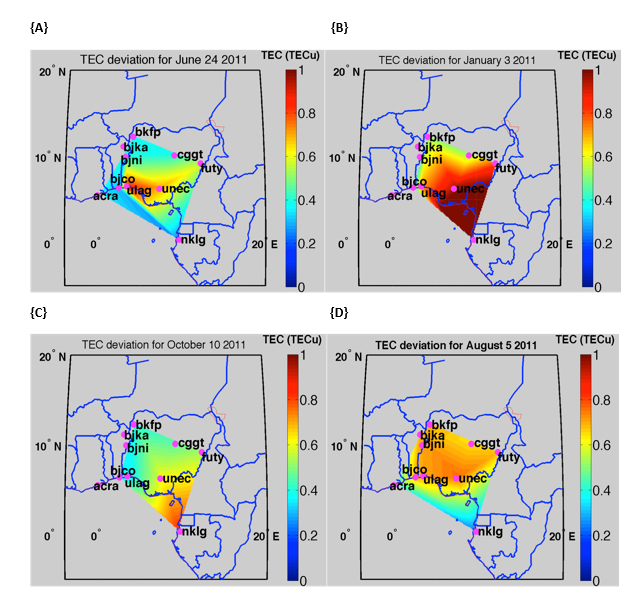


Fig. S3 Extrapolated contour plot of the absolute peak TEC deviation magnitude showing the direction of propagation of the resulting gravity wave for the thunderstorm event (A) at Lagos on the 24^th^ of June, (B) at Libreville on the 3^rd^ of January, (C) at Libreville on the 10th of October, 2011 and (D) at Enugu on the 5^th^ of August, 2011.


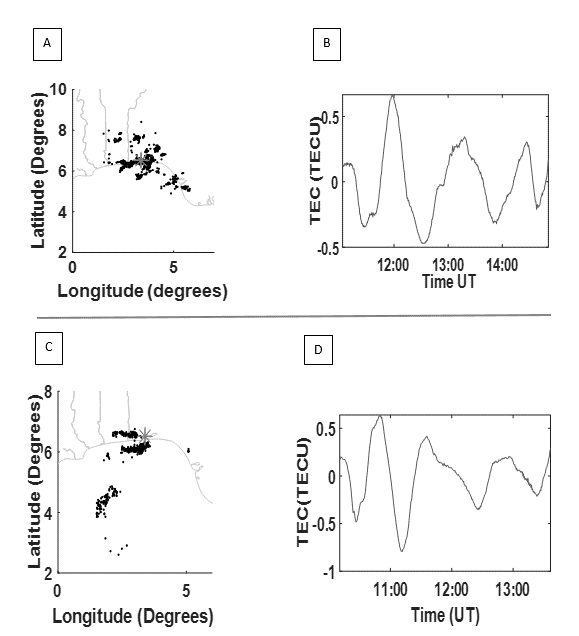


Fig. S4 Intensified lightning around Lagos within 200 km radius with corresponding TEC deviation. **Upper panel:** for Lagos on 24th of June. **Lower Panel:** for Lagos on the 8th may


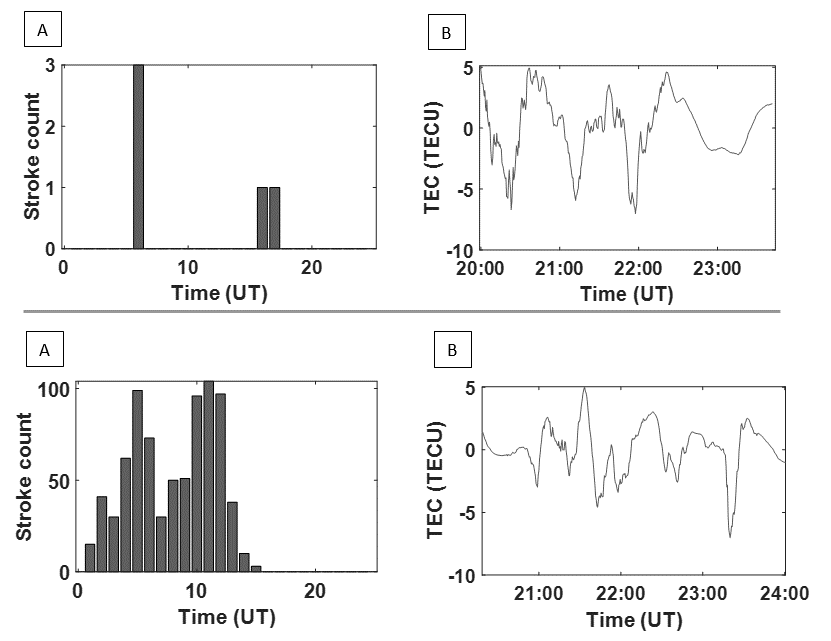


Fig. S5 **Upper panel** (A) Stroke count for April 27, 2011 indication a presence of lightning and thunderstorm event; (B) the corresponding post sunset Night time TEC deviation, which is enhanced without Thunderstorm. **Lower Panel** (A) stroke count for May 14, 2011 indication absence of flash count at post sunset; (B) the corresponding post sunset TEC deviation, which is enhanced without Thunderstorm.

Table S1 Days without thunderstorm in 2011 with low values of TEC deviation (absolute peak values) which are mostly below 0.3 TECUs

| January | PRN 6 | PRN 11 | PRN 20 | PRN 32 |
| --- | --- | --- | --- | --- |
| 8-Jan | 0.2717 | 0.145 | 0.1746 | 0.1349 |
| 9-Jan | 0.176 | 0.1853 | 0.2315 | 0.1864 |
| 10-Jan | 0.205 | 0.2236 | 0.2099 | 0.2274 |
| 11-Jan | 0.2012 | 0.2098 | 0.1558 | 0.1832 |
| 13-Jan | 0.1511 | 0.2298 | 0.1152 | 0.1852 |
| 17-Jan | 0.184 | 0.2928 | 0.2537 | 0.3375 |
| 18-Jan | 0.2928 | 0.1272 | 0.2954 | 0.1932 |
| 19-Jan | 0.1718 | ND | 0.1909 | 0.2618 |
| 20-Jan | 0.1456 | 0.1913 | 0.1566 | 0.1836 |
| 23-Jan | 0.2302 | 0.1248 | 0.1592 | 0.268 |
| 24-Jan | 0.1856 | 0.1856 | 0.2556 | 0.2587 |
| 25-Jan | 0.1925 | 0.1396 | 0.1841 | 0.2279 |
| 27-Jan | 0.2645 | 0.1726 | 0.2478 | 0.3288 |
| 28-Jan | 0.1308 | 0.4087 | 0.1719 | 0.3499 |
| 30-Jan | 0.1886 | 0.2022 | 0.1344 | 0.2068 |
| February | PRN 11 | PRN 17 | PRN 19 | PRN 23 |
| 6-Feb | 0.3123 | 0.1074 | 0.1859 | 0.2481 |
| 7-Feb | 0.2461 | 0.0796 | 0.2875 | ND |
| 16-Feb | 0.2691 | 0.1247 | 0.1817 | 0.2821 |
| 28-Feb | 0.2308 | 0.1783 | 0.1979 | 0.1902 |

Tables S2 Values of TEC deviation during Daytime thunderstorm in 2011 with values of TEC deviation (absolute peak values) as high as 0.5-0.9 TECUs

| September | PRN 9 | PRN 16 | PRN 21 | PRN 22 | PRN 29 |
| --- | --- | --- | --- | --- | --- |
| 1-Sep | 0.274 | 0.7591 | 0.2963 | 0.5096 | 0.3996 |
| 4-Sep | 0.3372 | 0.2749 | 0.9031 | 0.6066 | 0.4847 |
| 9-Sep | 0.4884 | 0.3315 | 0.2449 | 0.3484 | 0.4091 |
| 11-Sep | 0.394 | 0.3862 | 0.6696 | 0.5257 | 0.4478 |
| 13-Sep | 0.416 | 0.1507 | 0.7765 | 0.3489 | 0.4904 |
| 17-Sep | 0.2094 | 0.6238 | 0.5155 | 0.4108 | 0.7701 |
| 18-Sep | 0.4321 | 0.2988 | 0.9476 | 0.5797 | 0.7107 |
| 19-Sep | 0.517 | 0.3812 | 0.6473 | 0.2663 | 0.576 |

Tables S3 Major daytime Thunderstorm days with high energy geomagnetic activities in September with suppressed TEC deviation values (absolute peak values)

| September | PRN 9 | PRN 16 | PRN 21 | PRN 22 | PRN 29 |
| --- | --- | --- | --- | --- | --- |
| 24-Sep | 0.2441 | 0.3113 | 0.3531 | 0.3867 | 0.6644 |
| 26-Sep | 0.3455 | 0.4932 | 0.4739 | 0.3347 | 0.464 |
| 27-Sep | 0.4034 | 0.4028 | 0.4275 | 0.3501 | 0.3227 |

Fig. S6 Time Series plots of TEC deviation obtained at Lagos for days without thunderstorm in January 2011 for satellite PRN 6 with undefined TEC deviation Trends

Fig. S7 Time Series plots of TEC deviation obtained at Lagos for days without thunderstorm in January 2011 for satellite PRN 20 with undefined TEC deviation Trends.

Fig. S8 Time Series plots of TEC deviation obtained at Lagos for days without thunderstorm in February 2011 for satellite PRN 17 with undefined TEC deviation Trends
